# The impact of hepatocellular carcinoma diagnosis on patients' health‐related quality of life

**DOI:** 10.1002/cam4.4166

**Published:** 2021-08-18

**Authors:** Manisha Verma, James M. Paik, Issah Younossi, Daisong Tan, Hala Abdelaal, Zobair M. Younossi

**Affiliations:** ^1^ Betty and Guy Beatty Center for Integrated Research Inova Health System Falls Church Virginia USA; ^2^ Center for Liver Disease Department of Medicine Inova Fairfax Medical Campus Falls Church Virginia USA; ^3^ Inova Medicine Inova Health System Falls Church Virginia USA; ^4^ Center for Outcomes Research in Liver Diseases Washington DC USA

**Keywords:** hepatocellular carcinoma, quality of life

## Abstract

**Background:**

Patients with hepatocellular cancer (HCC) are known to have worse health‐related quality of life (HRQL) than the general population. However, the change in HRQL from before the diagnosis to after diagnosis remains unknown and is difficult to estimate. We aimed to compare HCC cases with matched controls to evaluate the differences in change in HRQL from before to after HCC diagnosis.

**Methods:**

We performed propensity score‐matched analysis using the self‐reported HRQL data from the Surveillance, Epidemiology, and End Results registries (SEER) data linked with Medicare Health Outcomes Survey (MHOS) data (1998–2014). Cases were selected as Medicare beneficiaries (aged ≥65 years) who were diagnosed with HCC between their baseline assessment and follow‐up assessment. Matched controls were selected from the same data resource and the same time period to include subjects without cancer diagnosis by propensity scores. HRQL was assessed using the Medical Outcomes Study Short Form‐36 (SF‐36).

**Results:**

The study included 62 subjects who developed HCC and 365 matched controls. Compared to their baseline HRQL scores, after diagnosis of HCC, subjects were more likely to report declines in scores related to the mental component of HRQL. When stratified by time since diagnosis, mental component remained significantly lower as the disease advanced. In contrast, only general health aspects of physical health worsened after HCC diagnosis.

**Conclusions:**

Diagnosis of HCC has a profound negative impact on patients’ HRQL. Mental health component deteriorated significantly over time. The need of including mental health services within a multidisciplinary HCC care model is clearly evident.

## Graphical abstract

Patients with hepatocellular cancer are known to have worse quality of life (QOL), and the change in QOL from before the diagnosis to after diagnosis remains unknown. We report the change in QOL post‐diagnosis, as this information can inform clinicians on what to expect.

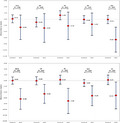

## 1 INTRODUCTION

Hepatocellular cancer (HCC) usually arises in the setting of an underlying progressive chronic liver disease, and affects millions of lives globally.^1^ It is the sixth most common cancer diagnosis and second leading cause of cancer‐related deaths worldwide, and is a major public health challenge.^2^ In the United States, HCC had more than 30,000 new cases in 2019, with men impacted two to three times more than women.^3^ It is projected that there will be more than 56,000 HCC cases in the United States by 2030.^4^ Notably, this is the only cancer for which the incidence and mortality rates have continued to rise exponentially over the past two decades.^5^, ^6^ Approximately one‐third of HCC cases are diagnosed at an advanced stage when curative treatment options are largely ineffective or not available.^7^ Due to the lethal nature of HCC, coupled with delayed diagnosis, the relative survival rate is below 30%.^8^ A recent systematic review on epidemiology, humanistic, and economic impact of HCC reported impaired Health‐Related Quality of Life (HRQL) linked with high direct costs and limited availability of treatment options.^9^ The expert panel on trial design for HCC recommended including HRQL as one of the outcomes when testing locoregional or systemic therapies.^10^

The clinical endpoints such as the risk of recurrence after treatments and mortality do not fully capture the spectrum of burden due to HCC. In this context, due to the impact of underlying chronic disease, delayed diagnosis, high symptom burden, and psychological distress, HRQL is reported to be severely diminished in HCC patients.^11^ However, there is limited data as to which particular aspects of HRQL are affected and what changes occur in HRQL after the diagnosis of HCC. Therefore, our aim was to assess the change in HRQL from before HCC diagnosis to after diagnosis using the Surveillance, Epidemiology, and End Results registries (SEER) data linked with Medicare Health Outcomes Survey (MHOS) data. We hypothesized that patients with HCC will show a decline in HRQL after diagnosis when compared with matched controls. This information will serve as an evidence base for future research and clinical interventions aimed at remediating these effects.

## 2 STUDY METHODS

### 2.1 Data sources

This study cohort was identified from the Surveillance, Epidemiology, and End Results (SEER)—Medicare Health Outcomes Survey (MHOS) data linked to population‐based data providing detailed information about older Americans with cancer. The MHOS, supported by the Centers for Medicare & Medicaid Services, is a yearly survey administered to a random sample of 1000 to 1200 Medicare beneficiaries from each managed care plan. The selected participants completed a survey at baseline and a 2‐year follow‐up survey if they still remained in the same plan. The SEER program, supported by the National Cancer Institute, collected information about patients with newly diagnosed cancer within specific geographic regions. A description of the SEER‐MHOS data resource has been published previously.^12^, ^13^

We included 15 MHOS cohorts with baseline assessments from the period 1998 to 2012 and follow‐up assessments from the period 2000 to 2014. Response rates on the MHOS baseline survey, linked to SEER data, ranged from 66.1% in 1998 to 51.6% in 2012.^14^ Follow‐up response rates ranged from 87.0% in 2000 to 70.3% in 2015, resulting in a sample size of 81,642 MHOS respondents linked to SEER across the 15 cohorts. The extent of potential nonresponse bias was studied previously, suggesting that response bias would be minimal in our study.^15^

To prospectively assess the impact of HCC diagnosis on HRQL, Medicare beneficiaries (aged ≥65 years) who developed HCC between the baseline and the follow‐up MHOS are included. We identified 62 patients with HCC who completed both baseline and the follow‐up MHOS. Controls were selected using propensity score‐matched analysis (5:1 control to case ratio) from the same data resource and the same period of time from subjects without cancer but completed both baseline and follow‐up MHOS. Matching was based on patient demographics, survey characteristics, and preexisting chronic medical conditions other than cancer. Our final study cohort included 365 control cases matched to 62 patients with HCC.

### 2.2 Data collection

The MHOS provides self‐reported data on sociodemographic, survey characteristics, chronic medical conditions, and HRQL. HRQL was assessed using the Medical Outcomes Study Short Form‐36 (SF‐36) from 1998 to 2005 and the Veterans RAND 12‐Item (VR‐12) from 2006 to 2014. The MHOS used the algorithm to recode the 8 VR‐12 scales to match the SF‐36.^16^ SF‐36/VR‐12 is a generic HRQL instrument designed to assess well‐being, functional status, and patient's perception of their health. It has eight subscales (Physical Functioning [PF], Role‐Physical [RP], Bodily Pain [BP], General Health [GH], Vitality [VT], Social Functioning [SF], Mental Health [MH], Role‐Emotional [RE]), two summary scores: Physical Component Summary (PCS) and Mental Component Summary (MCS). Each of the eight scale measures as well as two summary scales was transformed to a T‐score metric, normed to the general the U.S. population, with a mean of 50, standard deviation of 10, and range of 0–100. Higher scores indicate better health. The minimal clinically important difference of 2 for summary measures and 4 for subscales was utilized.^17^

### 2.3 Statistical methods

We compared socio‐demographic characteristics, preexisting comorbid conditions. and HRQL measures at baseline between HCC cases and controls using a non‐parametric Kruskal–Wallis test for continuous variables and chi‐square test for categorical variables.

To investigate whether there is a difference in HRQL after diagnosis of HCC, the longitudinal mixed models based on generalized estimating equations (GEE) were performed, allowing the adjustments for socio‐demographic characteristics as well as time‐variant covariates between surveys. The models included time‐invariant covariates (sex, race, and education) as well as time‐variant covariates: age, assessment mode, Medicaid, active smoker, and self‐reported comorbid conditions (hypertension, heart disease, stroke, Crohn's disease, arthritis, sciatica, diabetes, and depression). Changes in HRQL measures between surveys were tested by the use of orthogonal contrasts using the fully adjusted model. The adjusted estimates of HRQL measures are reported in this paper since similar findings were observed in unadjusted estimates.

To understand the effect of time since HCC diagnosis on HRQL measures, time since diagnosis was categorized into two groups: time since diagnosis of ≤12 months and >12 months. Univariable logistic regression is applied for evaluating predictors of a meaningful decrease in HRQL. All analyses were performed using SAS version 9.4 (SAS Institute Inc., Cary, NC).

## 3 RESULTS

### 3.1 Baseline characteristics

Demographics characteristics and comorbidities for the HCC cases and the matched non‐cancer controls were compared (Table 1). The mean (SD) age of the entire study population is 72.57 (9.19) years, 57% were Caucasians, and 40% were females. In the study group, 61% represented the western region of the United States, and 12% each from the northeast, Midwest, and eastern regions. 73% of the entire cohort had a history of hypertension, 60% had arthritis, and 26% had heart disease. 40% had a history of diabetes and 40% with depression diagnosis. 85% of surveys were assessed using the mailed method and remaining by phone. There were no significant differences between the two groups on demographics or comorbidities because of the propensity score matching (*p *> 0.05). The median (Interquartile range) time between surveys was 25.1 (23.8 to 26.5) months for HCC cases and 25.3 (24.4 to 27.1) months for matched controls.

**TABLE 1:** Baseline characteristics for hepatocellular cancer cases and matched non‐cancer controls

Characteristic	Hepatocellular cancer (*n* = 62)	Non‐cancer controls (*n* = 365)	*p*
Age	71.70 ± 7.17	72.71 ± 9.49	0.3260
Female	20 (33.33%)	151 (41.37%)	0.2394
Race			
White	30 (53.57%)	210 (57.53%)	0.5770
Black	<11 (<18.0%)	23 (6.30%)	0.7846
Hispanic	12 (21.43%)	77 (21.10%)	0.9547
College degree	22 (36.07%)	119 (32.60%)	0.5947
Married	36 (61.02%)	200 (54.79%)	0.3720
Active smoker	<11 (<18.0%)	47 (12.88%)	0.4863
Assessment mode			
Mailed	53 (85.48%)	311 (85.21%)	0.9544
Telephone	<11 (<18.0%)	54 (14.79%)	0.9544
Medicaid	12 (19.35%)	62 (16.99%)	0.6487
Region			
Northeast	<11 (<18.0%)	46 (12.60%)	0.9475
Midwest	<11 (<18.0%)	46 (12.60%)	0.5149
South	<11 (<18.0%)	43 (11.78%)	0.5426
West	39 (62.90%)	222 (60.82%)	0.7559
Comorbidities			
Hypertension	44 (73.33%)	264 (72.33%)	0.8717
Heart disease	18 (29.51%)	93 (25.48%)	0.5070
Stroke	<11 (<18.0%)	30 (8.22%)	0.6812
Crohn disease, ulcerative colitis or inflammatory bowel disease	<11 (<18.0%)	19 (5.21%)	0.9039
Arthritis	32 (51.61%)	222 (60.82%)	0.1721
Sciatica	23 (37.10%)	109 (29.86%)	0.2545
Diabetes	26 (41.94%)	145 (39.73%)	0.7427
Depression	21 (35.59%)	147 (40.27%)	0.4952

Note

Propensity Score Matching was based on patient demographics, survey characteristics, and chronic medical conditions other than cancer.

### 3.2 Baseline SF‐36 survey results

The adjusted mean of PCS and MCS for HCC cases and controls are presented in Table 2. There were no differences in the adjusted mean PCS between HCC cases and controls (44.4 [95% CI, 33.9 to 54.8] vs. 44.8 [34.8 to 54.7]) and MCS (53.7 [45.7 to 61.7] vs. 51.6 [44.0 to 59.2]), all *p*‐values >0.50 at baseline. The same result was observed for the eight subscale measurements. These baseline means are consistent with the 1988 U.S. population norms for adults aged 65 years or over (PCS, males = 42.0 and females = 41.0; and MCS, males = 52.5 and females = 51.4).^18^ Similar patterns were observed in the unadjusted SF‐36 scores at baseline (Table S1).

**TABLE 2:** Adjusted health‐related quality of life of baseline and follow‐up of patients with hepatocellular cancer and non‐cancer controls

	Hepatocellular cancer			Control			
	Baseline mean (95% CI)	Follow‐up mean (95% CI)	*p* *	Baseline mean (95% CI)	Follow‐up mean (95% CI)	*p* *	*p* **
PCS	44.37 (33.92–54.81)	41.74 (31.24–52.23)	0.1941	44.75 (34.77–54.73)	44.86 (34.61–55.12)	0.8648	0.1981
Bodily pain	40.43 (31.92–48.95)	38.79 (29.72–47.86)	0.3765	40.92 (32.57–49.27)	40.83 (32.22–49.44)	0.8846	0.4299
Physical function	45.81 (34.26–57.36)	42.94 (30.79–55.10)	0.2283	46.50 (35.48–57.53)	45.51 (34.09–56.93)	0.2040	0.4528
Role physical	48.83 (39.07–58.59)	46.67 (36.62–56.73)	0.3420	49.14 (39.77–58.52)	50.42 (40.76–60.08)	0.0900	0.1500
General health	47.70 (37.61–57.80)	41.08 (30.81–51.35)	0.0013	46.53 (37.09–55.97)	46.42 (36.71–56.14)	0.8783	0.0030
MCS	53.71 (45.70–61.73)	47.35 (39.03–55.68)	0.0005	51.60 (44.00–59.19)	51.62 (43.88–59.36)	0.9732	0.0010
Mental health	51.28 (43.09–59.46)	46.03 (37.37–54.70)	0.0029	49.65 (41.77–57.53)	48.83 (40.79–56.88)	0.1968	0.0175
Role emotional	51.02 (41.39–60.65)	45.89 (35.93–55.84)	0.0313	49.91 (40.95–58.87)	50.17 (40.90–59.45)	0.7553	0.0329
Social function	53.77 (43.80–63.75)	46.73 (36.53–56.92)	0.0016	52.06 (42.74–61.39)	52.39 (42.70–62.07)	0.6846	0.0019
Vitality	49.62 (40.52–58.73)	45.03 (35.82–54.23)	0.0059	49.16 (40.55–57.78)	49.12 (40.14–58.09)	0.9480	0.0129

Note

Adjustments for patient demographics, survey characteristics, and chronic medical conditions other than cancer.

Abbreviations: CI, confidence interval; MCS, mental component summary; PCS, physical component summary.

* *p*‐value for change between baseline and follow‐up.

** *p*‐value for differences in change from baseline to follow‐up between HCC cancer cases and non‐cancer controls.

### 3.3 Change in HRQL over time (cases vs. controls)

**FIGURE 1:**
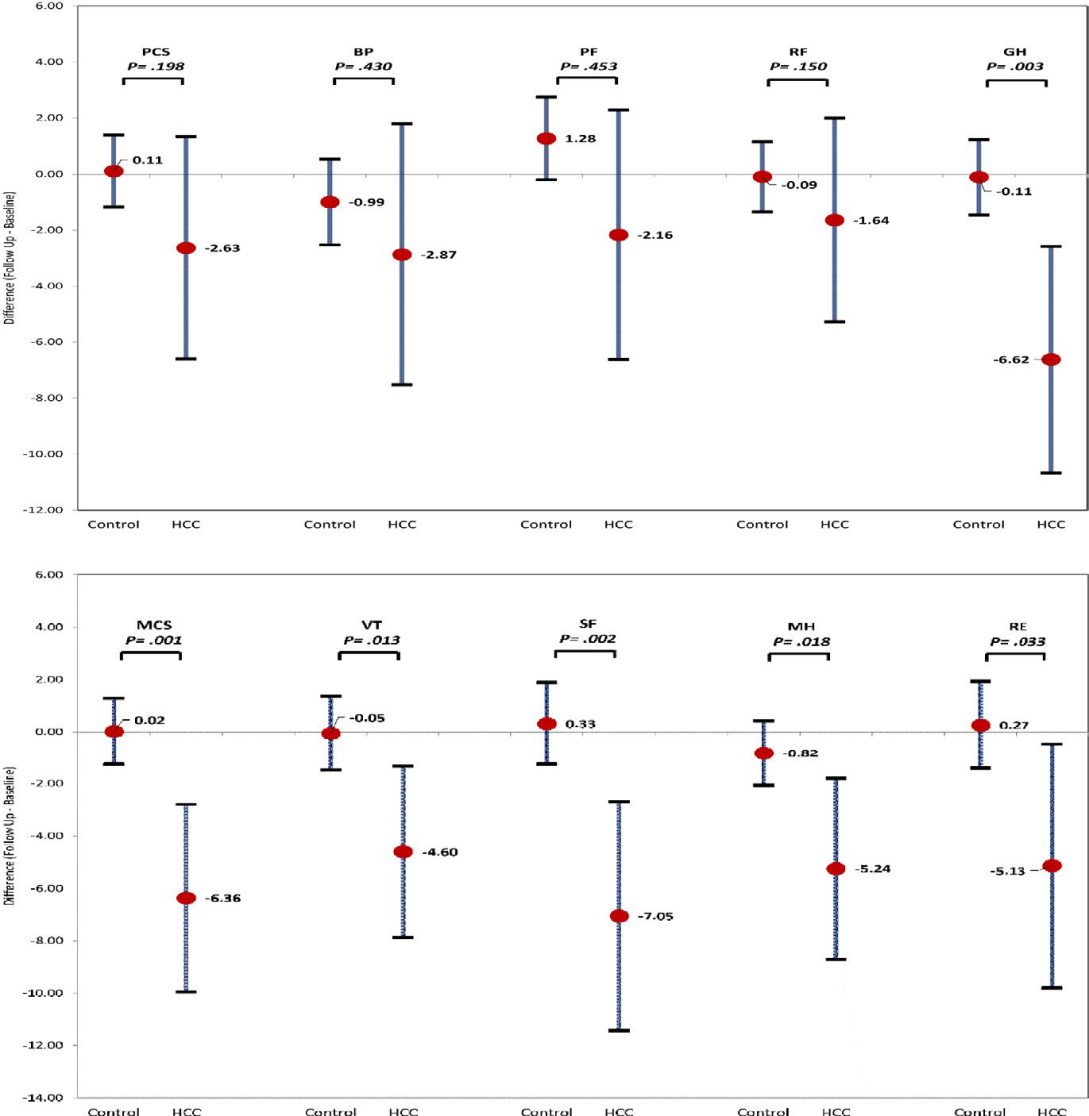
Adjusted mean differences in the PCS and MCS between baseline and follow‐up assessment for HCC cases and non‐cancer controls. *p*‐value for differences in change from baseline to follow‐up between HCC cancer cases and non‐cancer controls. BP, bodily pain; GH, general health; MCS, mental component summary; MH, mental health; PCS, physical component summary; PF, physical function; RE, role emotional; RP, role physical; SF, social function; VT, vitality

Compared to their baseline HRQL scores, after diagnosis of HCC, subjects were more likely to report declines in PCS and MCS as compared to non‐cancer controls (mean decline = −2.63 [−6.60 to 1.34] vs. 0.11 [−1.17 to 1.39], *p *= 0.198 and −6.36 [−9.94 to −2.77] and 0.02 [−1.25 to 1.29], *p *= 0.001, respectively) (Figure 1). These trends differed substantially across the eight subscale measures. Compared to the controls, the greatest declines after diagnosis of HCC were observed in social function/SF (mean decline = −7.05 [−11.41 to −2.68] vs. 0.33 [−1.24 to 1.89] *p *= 0.002), followed by general health/GH (−6.62 [−10.66 to −2.58] vs. −0.11[−1.45 to 1.24], *p *= 0.003), mental health/MH (−5.24 [−8.69 to −1.79] vs. −0.82 [−2.05 to 0.42], *p *= 0.018), role emotional/RE (−5.13 [−9.80 to −0.46] vs. 0.27 [−1.41 to 1.94], *p *= 0.033), and vitality/VT (−4.60 [−7.87 to −1.32] vs. −0.05 [−1.47 to 1.37], *p *= 0.013) scores (Table 2 and Figure 1). In contrast, no significant changes were noted in physical function/PF, role physical/RP, and bodily pain/BP (−2.87 [−7.53 to 1.80] vs. −0.99 [−2.52 to 0.54]; −2.16 [−6.62 to 2.30] vs. 1.28 [−0.20 to 2.75]; and −1.64 [−5.28 to 2.00] vs. −0.09 [−2.52 to 0.54], all *p *> 0.100). Notably, 52% HCC patients had more than 4‐point decrease in PCS score and 61% on MCS (Table S2). This reflects a clinically meaningful decrease in score from baseline to follow‐up. In the unadjusted model, HCC patients with diabetes and depression were more likely to have clinically meaningful declines in MCS (Odd ratio = 1.94 [0.60–6.31] and 4.02 [1.07–15.07]) (Data not shown). Given the small sample size, multivariable analysis was not considered.

### 3.4 Change in HRQL over time by time since diagnosis and by stages Among HCC cases

The median time from HCC diagnosis to follow‐up completion of the survey was 8.5 months (Interquartile range, IQR [5 to 16 months]) (Tables 3 and 4).

**TABLE 3:** Adjusted change in health‐related quality of life from before to after hepatocellular cancer diagnosis, by time since diagnosis

	Time since diagnosis 0–12 months (*n* = 37)	Time since diagnosis ≥12 months (*n* = 25)	*P*
PCS	−4.39 (−9.50 to 0.72)	−0.33 (−7.36 to 6.69)	0.3955
Bodily pain	−1.35 (−12.90 to 10.21)	−6.96 (−19.10 to 5.17)	0.5177
Physical functioning	−7.52 (−20.89 to 5.85)	−5.64 (−23.43 to 12.16)	0.8774
Role physical	−2.09 (−7.52 to 3.35)	−1.44 (−8.73 to 5.85)	0.8916
General health	−7.32 (−12.06 to −2.58)	−6.21 (−12.66 to 0.23)	0.8013
MCS	−0.24 (−4.72 to 4.25)	−10.16 (−14.66 to −5.66)	0.0055
Mental health	0.53 (−3.77 to 4.83)	−8.03 (−12.16 to −3.91)	0.0047
Role emotional	−2.35 (−9.06 to 4.36)	−7.98 (−13.73 to −2.22)	0.2448
Social functioning	−4.31 (−11.02 to 2.41)	−7.79 (−11.81 to −3.76)	0.4109
Vitality	−1.72 (−5.75 to 2.31)	−6.08 (−11.01 to −1.14)	0.1922

Note

Data display as adjusted change (95% confidence interval).

Adjustments for patient demographics, survey characteristics, and chronic medical conditions.

*p* value for differences in change from baseline to follow‐up between HCC patients with time since diagnosis <12 months and ≥12 months.

**TABLE 4:** Adjusted change in health‐related quality of life of baseline and follow‐up of patients with hepatocellular cancer, by the AJCC stage

	Hepatocellular cancer		
	Stage 1 or 2 (*n* = 33)	Stage 3 or 4 (*n* = 20)	*p*
PCS	−0.49 (−5.77 to 4.78)	−2.47 (−11.79 to 6.86)	0.7202
Bodily pain	−0.26 (−5.39 to 4.87)	−4.59 (−10.82 to 1.65)	0.2943
Physical functioning	0.43 (−5.24 to 6.10)	−4.09 (−13.14 to 4.95)	0.4140
Role physical	−1.39 (−7.15 to 4.37)	2.50 (−4.29 to 9.28)	0.3891
General health	−4.95 (−10.00 to 0.09)	−7.50 (−14.77 to −0.22)	0.6023
MCS	−3.44 (−7.39 to 0.51)	−6.23 (−12.01 to −0.45)	0.4337
Mental health	−0.88 (−5.10 to 3.35)	−6.54 (−12.46 to −0.62)	0.1152
Role emotional	−5.78 (−12.01 to 0.45)	−4.75 (−12.04 to 2.54)	0.8315
Social functioning	−2.78 (−7.81 to 2.25)	−6.17 (−11.02 to −1.31)	0.3510
Vitality	0.33 (−4.16 to 4.81)	−6.65 (−10.71 to −2.60)	0.0173

Note

Data display as adjusted change (95% confidence interval).

Adjustments for patient demographics, survey characteristics, and chronic medical conditions.

*p* value for differences in change from baseline to follow‐up between HCC patients with Stage 1 or 2 and Stage 3 or 4.

Of the 62 HCC cases, although there is no statistical difference on the mean change in PCS as well as BP, PF, RP, and GH score by time since diagnosis (*p *> 0.400), numerically higher decline for physical health scales in HCC patients within 1 year of diagnoses versus post 1 year of diagnosis for all domains except bodily pain was found (PCS, −4.39 vs. −0.33; PF, −7.52 vs. −5.64; RP −2.09 vs. −1.44; and GH, −7.32 vs. −6.21). In contrast, the adjusted mean declines in MCS and MH score among HCC patients within 1 year of diagnosis and post 1 year of diagnosis were both clinically and statistically significant (MCS −0.24 [−4.72 to 4.25] vs. −10.16 [−14.66 to −5.66], *p* < 0.001; MH (0.53 [−3.77 to 4.83] and −8.03 [−12.16 to −3.91], *p* < 0.005). This clearly shows that the negative effects of HCC for all the physical health scales, except bodily pain, stabilized over time, whereas those on all the mental health scales continued to progressively decline over time. Staging was defined using Stage derived from the American Joint Committee on Cancer (AJCC) staging system. Of the 53 HCC cases (with AJCC stage available), compared to HCC patients with stage 1 or 2, HCC patients with stage 3 or 4 had no statistical difference but numerically higher adjusted mean declines in PCS (−0.49 vs. −2.47); RF (−0.26 vs. −4.59); GH (−4.95 vs. −7.5); MCS (−3.44 vs. −6.23); MH (−0.88 vs. −6.54); and SF (−2.78 vs. −6.17). In contrast, the adjusted mean declines in VT scores between stage 1 or 2 and stage 3 or 4 were both clinically and statistically significant (0.33 [−4.16 to 4.81] vs. −6.65 [−10.71 to −2.60], *p* = 0.017) (Table 4). The unadjusted mean HRQL scores by the AJCC stage are reported in Table S3.

## 4 DISCUSSION

Diagnosis of HCC in itself is associated with poor prognosis and negative impact on patients’ clinical outcomes and survival.^1^ HRQL has been reported to serve as a prognostic marker for HCC survival.^19^, ^20^ Patients with HCC are known to suffer from impaired HRQL when compared to the general population or those with chronic liver disease alone.^21^, ^22^, ^23^ In fact, a study has shown that patients with HCC had worse global HRQL, physical function, role, cognitive, and social function.^24^ Another recent study found a self‐reported reduction in ability to concentrate in 47%, reduced physical function in 44%, and diminished overall mental health in 36% of HCC patients, when asked for self‐reflect on their condition before HCC diagnosis.^25^ Our data provide more specific details about components of HRQL that worsen over time, notably when compared to those before diagnosis. Particularly, our data show that the mental health component of HRQL is significantly affected by the diagnosis of HCC and this impairment worsens significantly over time. In the first 12 months after diagnosis, physical health domains such as general health and physical function are impacted. However, after the first 12 months of diagnosis, these aspects of HRQL either improve or remain stable. In contrast, most components of mental health seem to be less profoundly impacted in the first 12 months but the trends continue to worsen over time. Patients with AJCC stage >2 had a worse decline in physical and mental health, with a clinical and statistically significant decline in vitality scores.

These findings have important clinical implications. It is important to provide clinical strategies that could aim for improvement in both physical and mental health aspects of HRQL in the first‐year post‐HCC diagnosis. Moreover, more long‐term strategies are needed to address mental health impairment post 1 year. These could include mental health services within multidisciplinary HCC clinics, or even social work consults who can help align resources such as linkage with psychotherapy or psychologists. Holistic approaches such as palliative care, which aim for both physical and mental health assessment and management may also prove beneficial, but unfortunately are underutilized for HCC patients.^26^ As demonstrated for lung cancer patients, early palliative care can help improve HRQL and depression over time.^27^

To address the mental health component, patient advocacy organizations can also serve a role, and offer resources to ease the distress and offer support. Notably, similar results of the declined mental component of HRQL scores were reported in a study comparing older adults with and without cancer using the same database.^28^ They projected this decline to be linked with psychosocial domains of health.

Above all, HRQL is critically influenced by time since diagnosis, probably due to increased stress given limited treatment options and dismal prognosis; hence the needs may vary over time. Patients with advanced stages have a worse decline in HRQL over time. Prospective studies are needed to evaluate the changes in HRQL domains over time, possibly through long‐term HCC registries enrolling patients across the country. Our international scientific societies could potentially lead hosting such registries and make them universally available for HCC patients using multimedia campaigns. However, these registries require the inclusion of appropriate HRQL instruments with good psychometric characteristics able to detect changes over time and capture the stage of HCC and ongoing treatments simultaneously to allow for structured analyses.

A diagnosis of cancer influences different aspects of lives including psychological, social, and emotional, physical indirectly or directly impacting HRQL.^29^ To our knowledge, this is the first study to assess changes in HRQL after HCC diagnosis, compared with a matched non‐cancer control group. The strengths of this study include the selection of HCC cases for which we had baseline and follow‐up data available, and identifying matched controls (particularly matched on medical history and age).

There are a number of limitations to the study. First, HCC or liver‐specific measures of HRQL are not available in the SEER‐MHOS data resource, which limits a comprehensive understanding of the impact of HCC. Although the SF‐36 is the most widely used instrument to assess overall HRQL, its limitation is its high ceiling effects which may not allow it to detect improvement or deterioration over time. Additionally, all HRQL data have a potential bias (reporter bias) and other social confounding factors which cannot be completely controlled for. Mortality data are not available, which limits the estimation of the prognostic value of HRQL and its change over time.

Second, SEER‐MHOS participants are enrolled in Medicare managed care, not fee‐for‐service Medicare beneficiaries. Thus, this data resource was not linked with Medicare billing claims. Due to this, the data resource does not contain any treatment or comorbidity information beyond self‐reported, which are covariates impacting HRQL. In addition, this data may not be representative of Medicare fee‐for‐service. Evidence of health status between managed care and Medicare fee‐for‐service beneficiaries is mixed across previous studies.^30^, ^31^, ^32^ Furthermore, patients with HCC who died before their follow‐up survey or disenrolled from the plan were not included, leading to the effect of response bias which is hard to estimate using this data resource. Third, because of the need for both baseline and follow‐up HRQL assessments for the objective of our study, the sample size was ultimately quite reduced, leading that the power to detect important changes in HRQL was reduced and multivariable data analyses were limited. In addition, the small sample size for staging may not capture the actual effect of staging on QoL among patients with HCC.

In summary, our study showed that HCC diagnosis negatively impacts HRQL. Both physical and mental health are affected. We found that the HRQL of HCC patients is worse than the general population, consistent with the literature. Over time, mental health becomes worse and needs more attention, potentially due to the effects of malignancy, as seen in other cancers. Given the high incidence and mortality of HCC cases, the need to address HRQL issues is urgent. Future research needs to evaluate the value of HRQL scores in comparative effectiveness research involving therapeutic options and psychological interventions.

## ETHICAL APPROVAL STATEMENT

The study was considered exempt by the Institutional IRB. The manuscript has been reviewed and approved by the National Cancer Institute, Outcomes Research Branch as a part of the SEER‐MOHS review process.

## CONFLICT OF INTEREST

ZMY has received research funds or served as a consultant to Gilead Sciences, Intercept, Novo Nordisk, BMS, Abbvie, Merck, Madrigal, Genfit, Siemens, BMS, Terns, and Viking. All other authors have no conflict of interest to disclose.

## AUTHOR CONTRIBUTIONS

Study design: James M. Paik, Zobair Younossi, Manisha Verma. Data collection: James M. Paik. Data analysis: James M. Paik. Interpretation of data: Manisha Verma, James M. Paik, Zobair M. Younossi. Drafting of the manuscript: Manisha Verma, James M. Paik, Issah Younossi, Daisong Tan, Hala Abdelaal, Zobair M. Younossi. Critical revision of the manuscript for important intellectual content: Manisha Verma, James M. Paik, Zobair M. Younossi. Study supervision: Zobair M. Younossi. All authors read and approved the final version of the manuscript.

## Supporting information

Table S1‐S3

## Data Availability

The SEER‐MHOS data that support the findings of this study are not openly available. Access to the de‐identified SEER‐MHOS dataset requires approval from the National Cancer Institute. Required documents and Instructions for obtaining the SEER‐MHOS dataset can be found at https://healthcaredelivery.cancer.gov/seer‐mhos/obtain/req.docs.html.
