# Supplementary material for: The impact of hepatocellular carcinoma diagnosis on patients' health‐related quality of life
Source: Cancer Med. 2021 Aug 18;10(18):6273–81. doi: 10.1002/cam4.4166 (PMC8446553; doi:10.1002/cam4.4166)
Supplement: Supplementary file 1 — Table S1‐S3 [file CAM4-10-6273-s001.docx]

| SUPPLEMENTARY TABLE 1. Unadjusted Health-related Quality of Life of baseline and follow-up of Patients with Hepatocellular Cancer, by Time since diagnosis | | | | |
| --- | --- | --- | --- | --- |
|  | Hepatocellular Cancer | | | |
|  | Total Sample of HCC Cancer (n=62) | Time since diagnosis 0-12 months (n=37) | Time since diagnosis ≥12 months (n=25) | P |
| Base line | | | | |
| PCS | 33.38 ± 12.70 | 33.24 ± 12.94 | 33.57 ± 12.62 | 0.8621 |
| Bodily Pain | 39.21 ± 11.15 | 38.59 ± 11.38 | 40.09 ± 10.98 | 0.4694 |
| Physical Functioning | 33.79 ± 12.36 | 33.94 ± 12.15 | 33.56 ± 12.90 | 0.8513 |
| Role Physical | 35.91 ± 12.12 | 35.98 ± 11.80 | 35.83 ± 12.82 | 0.2491 |
| General Health | 39.09 ± 12.95 | 39.62 ± 12.20 | 38.33 ± 14.19 | 0.6362 |
| MCS | 51.03 ± 11.15 | 50.46 ± 11.21 | 51.85 ± 11.26 | 0.6471 |
| Mental Health | 49.30 ± 10.47 | 48.97 ± 10.05 | 49.77 ± 11.24 | 0.6756 |
| Role Emotional | 45.17 ± 12.63 | 45.10 ± 12.99 | 45.27 ± 12.37 | 0.4934 |
| Social Functioning | 43.86 ± 12.19 | 44.71 ± 12.08 | 42.64 ± 12.48 | 0.7279 |
| Vitality | 43.30 ± 11.40 | 42.39 ± 11.96 | 44.64 ± 10.63 | 0.7095 |
| Follow-up | | | | |
| PCS | 31.20 ± 10.92 | 30.08 ± 11.17 | 32.84 ± 10.55 | 0.3159 |
| Bodily Pain | 37.26 ± 11.86 | 35.97 ± 12.03 | 39.26 ± 11.56 | 0.4388 |
| Physical Functioning | 31.28 ± 12.51 | 32.01 ± 12.83 | 30.21 ± 12.19 | 0.6481 |
| Role Physical | 34.04 ± 11.67 | 32.30 ± 10.67 | 36.61 ± 12.79 | 0.1817 |
| General Health | 34.40 ± 12.84 | 33.49 ± 13.60 | 35.75 ± 11.76 | 0.6033 |
| MCS | 45.77 ± 13.33 | 45.55 ± 11.86 | 46.08 ± 15.48 | 0.8290 |
| Mental Health | 45.89 ± 12.30 | 46.28 ± 11.52 | 45.31 ± 13.61 | 0.9713 |
| Role Emotional | 40.25 ± 13.73 | 39.74 ± 14.25 | 40.97 ± 13.23 | 0.7272 |
| Social Functioning | 36.04 ± 14.07 | 35.20 ± 14.57 | 37.29 ± 13.49 | 0.5231 |
| Vitality | 40.52 ± 11.73 | 40.28 ± 11.40 | 40.89 ± 12.42 | 0.9481 |
| Abbreviations: PCS, physical component summary; MCS, mental component summary; CI, confidence interval Data display as mean ± Standard deviation | | | | |

| SUPPLEMENTARY TABLE 2: Clinically Meaningful Differences in SF-36 scores (from baseline to follow-up) of Patients with Hepatocellular Cancer (N=62) | | | |
| --- | --- | --- | --- |
|  |  |  | count (%) |
| PCS | Decrease > 4 points | PCS | 29 (51.79) |
| Bodily Pain | Decrease > 2 points | Bodily Pain | 18 (29.51) |
| Physical Functioning | Decrease > 2 points | Physical Functioning | 26 (41.94) |
| Role Physical | Decrease > 2 points | Role Physical | 24 (39.34) |
| General Health | Decrease > 2 points | General Health | 23 (37.70) |
| MCS | Decrease > 4 points | MCS | 34 (60.71) |
| Mental Health | Decrease > 2 points | Mental Health | 29 (47.54) |
| Role Emotional | Decrease > 2 points | Role Emotional | 25 (42.37) |
| Social Functioning | Decrease > 2 points | Social Functioning | 30 (49.18) |
| Vitality | Decrease > 2 points | Vitality | 30 (50.85) |
| Abbreviations: PCS, physical component summary; MCS, mental component summary | | | |

| SUPPLEMENTARY TABLE 3. Unadjusted Health-related Quality of Life of baseline and follow-up of Patients with Hepatocellular Cancer, by Historical Stage | | | |
| --- | --- | --- | --- |
|  | Hepatocellular Cancer | | |
|  | Stage 1 or 2  (n=33) | Stage 3 or 4  (n=20) | P |
| Base line | | | |
| PCS | 30.38 ± 11.17 | 35.86 ± 12.79 | 0.1103 |
| Bodily Pain | 36.93 ± 10.87 | 41.88 ± 10.60 | 0.0491 |
| Physical Functioning | 29.87 ± 11.51 | 37.76 ± 10.92 | 0.0160 |
| Role Physical | 34.09 ± 11.17 | 35.85 ± 12.00 | 0.6049 |
| General Health | 37.49 ± 13.11 | 40.82 ± 13.47 | 0.3271 |
| MCS | 50.84 ± 11.98 | 49.79 ± 10.43 | 0.8557 |
| Mental Health | 47.60 ± 11.61 | 51.03 ± 8.99 | 0.3712 |
| Role Emotional | 44.99 ± 13.10 | 42.85 ± 13.20 | 0.2325 |
| Social Functioning | 41.28 ± 13.00 | 43.10 ± 10.46 | 0.5398 |
| Vitality | 41.67 ± 9.79 | 43.92 ± 13.13 | 0.3863 |
| Follow-up | | | |
| PCS | 29.35 ± 10.45 | 35.25 ± 10.53 | 0.0575 |
| Bodily Pain | 36.69 ± 12.31 | 37.38 ± 11.06 | 0.9094 |
| Physical Functioning | 29.21 ± 11.86 | 35.50 ± 13.27 | 0.0942 |
| Role Physical | 33.32 ± 11.00 | 35.78 ± 13.15 | 0.6054 |
| General Health | 34.05 ± 11.74 | 35.63 ± 14.59 | 0.5497 |
| MCS | 48.38 ± 14.49 | 41.46 ± 10.11 | 0.0356 |
| Mental Health | 47.09 ± 13.55 | 43.56 ± 9.21 | 0.1740 |
| Role Emotional | 41.81 ± 14.04 | 36.78 ± 13.97 | 0.1518 |
| Social Functioning | 36.90 ± 14.26 | 36.27 ± 13.21 | 0.7110 |
| Vitality | 42.13 ± 11.91 | 39.42 ± 10.84 | 0.5175 |
| Abbreviations: PCS, physical component summary; MCS, mental component summary; CI, confidence interval Data display as mean ± Standard deviation | | | |
